# Functional consequences of transferrin receptor-2 mutations causing hereditary hemochromatosis type 3

**DOI:** 10.1002/mgg3.136

**Published:** 2015-03-06

**Authors:** Ricky Joshi, Maya Shvartsman, Erica Morán, Sergi Lois, Jessica Aranda, Anna Barqué, Xavier de la Cruz, Miquel Bruguera, José Manuel Vagace, Guillermo Gervasini, Cristina Sanz, Mayka Sánchez

**Affiliations:** 1 Cancer and Iron Group and Advanced Genetic Diagnostic Unit of Rare Iron Disorders (UDGAEMH), Institut of Predictive and Personalized Medicine of Cancer (IMPPC)Barcelona, Spain; 2 Vall d'Hebron Research Institute (VHIR)Barcelona, Spain; 3 Institució Catalana de Recerca i Estudis Avançats (ICREA)Barcelona, Catalonia, Spain; 4 Service of Hepatology, Clinic Hospital of BarcelonaBarcelona, Spain; 5 Service of Haematology, Hospital Materno-Infantil de BadajozBadajoz, Spain; 6 Department of Surgical & Medical Therapeutics, University of ExtremaduraBadajoz, Spain; 7 Service of Haematology and Hemotherapy, Clinic Hospital of BarcelonaBarcelona, Spain; 8 Diagnostics in Iron Metabolism Service (D·IRON) and Iron Metabolism: Regulation and Diseases group, Josep Carreras Leukemia Research Institute (IJC)Barcelona, Spain

**Keywords:** Hereditary hemochromatosis type 3, iron overload, missense, nonsense, p.Gly792Arg, splicing mutation, *TFR2* gene

## Abstract

Hereditary hemochromatosis (HH) type 3 is an autosomal recessive disorder of iron metabolism characterized by excessive iron deposition in the liver and caused by mutations in the transferrin receptor 2 (*TFR2*) gene. Here, we describe three new HH type 3 Spanish families with four *TFR2* mutations (p.Gly792Arg, c.1606-8A>G, Gln306*, and Gln672*). The missense variation p.Gly792Arg was found in homozygosity in two adult patients of the same family, and in compound heterozygosity in an adult proband that also carries a novel intronic change (c.1606-8A>G). Two new nonsense *TFR2* mutations (Gln306* and Gln672*) were detected in a pediatric case. We examine the functional consequences of two *TFR2* variants (p.Gly792Arg and c.1606-8A>G) using molecular and computational methods. Cellular protein localization studies using immunofluorescence demonstrated that the plasma membrane localization of p.Gly792Arg TFR2 is impaired. Splicing studies in vitro and in vivo reveal that the c.1606-8A>G mutation leads to the creation of a new acceptor splice site and an aberrant TFR2 mRNA. The reported mutations caused HH type 3 by protein truncation, altering TFR2 membrane localization or by mRNA splicing defect, producing a nonfunctional TFR2 protein and a defective signaling transduction for hepcidin regulation. *TFR2* genotyping should be considered in adult but also in pediatric cases with early-onset of iron overload.

## Introduction

Iron is an essential nutrient with an indispensable biological role in numerous cellular processes. In the body, iron is tightly regulated, as both an excess and a deficiency lead to severe health complications (Hentze et al. 2010). Hereditary hemochromatosis (HH) is an autosomal recessive disorder characterized by excessive iron absorption and deposition in vital organs such as the liver, heart, and pancreas (Pietrangelo 2004). Without an early implementation of an adequate treatment, iron accumulation leads to tissue damage and clinical complications such as cirrhosis, diabetes mellitus, arthropathy, cardiomyopathy, hypogonadism, impotence, and hepatocellular carcinoma. Four types of HH are known and are attributed to genetic mutations in five different genes. The most common HH is the type 1 form (Online Mendelian Inheritance in Man, OMIM number #235200) caused by mutations in the major histocompatibility complex class I-like protein *HFE* (OMIM *613609) (Feder et al. 1996). HH type 2a and 2b (OMIM #602390 and #613313), also known as Juvenile hemochromatosis, are due to mutations in the *HFE2* (encoding hemojuvelin protein) and *HAMP* (encoding the iron hormone hepcidin) genes (OMIM *608374and *606464) (Roetto et al. 2003; Papanikolaou et al. 2004). In HH type 4 (OMIM #606069) patients have loss- or gain-of-function mutations in the iron exporter ferroportin that is encoded by the *SLC40A1* gene (OMIM *604653; Detivaud et al. 2013). HH type 3 (OMIM #604250) is a rare form of HH characterized by genetic alterations in the Transferrin receptor 2 (*TFR2*) gene (OMIM *604720; Camaschella et al. 2000). Although HH type 3 was initially described with adult onset, other patients have been reported with juvenile forms (Pietrangelo et al. 2005; Gerolami et al. 2008; Bardou-Jacquet et al. 2013).

The TFR2 protein is a transmembrane homodimer homolog of TFR1 and is mainly expressed in the liver (Kawabata et al. 1999). TFR2 has a major role in the regulation of iron homeostasis since it is required for adequate hepatic expression of the iron hormone hepcidin. Whole-body or liver-specific complete disruption of *TFR2* gene or point mutations in mice and rats recapitulated the human HH type 3 iron overload disease (Fleming et al. 2002; Wallace et al. 2005, 2007; Bartnikas et al. 2013). At the molecular level it is thought that TFR2 acts as a body iron sensor of diferric transferrin resulting in the upregulation of hepcidin production through a not yet fully understood signaling mechanism (West et al. 2000; Calzolari et al. 2006). In the cell surface TFR2 has been shown to colocalize with HFE in the duodenum (Griffiths and Cox 2003) and to interact with HJV in a hepatoma cell line (D'Alessio et al. 2012) to form a multiprotein complex needed for the hepcidin signaling. Recent studies indicate that the HFE–TFR2 interaction is not direct (Rishi et al. 2013).

Up to date, a total of 44 families (65 affected patients) with pathogenic mutations and suffering from HH type 3 have been described in the literature (Table S1). The murine counterparts of four human *TFR2* mutations (Met172Lys, AVAQ621_624del, Gln690Pro and Tyr250*) have been studied in a hepatic murine system (Hepa1-6 cells) for their consequence in TFR2 expression and localization. The authors conclude that *TFR2* mutations cause intracellular retention of the protein at the endoplasmic reticulum (Wallace et al. 2008).

Here, we describe four Spanish patients with HH type 3 and three novel mutations in the *TFR2* gene. In addition, we demonstrate that the p.Gly792Arg variation alone or in combination with a splicing defect (c.1606-8A>G) are causative pathogenic mutations for HH type 3.

## Material and Methods

### Patients

#### Family 1

**Figure 1:**
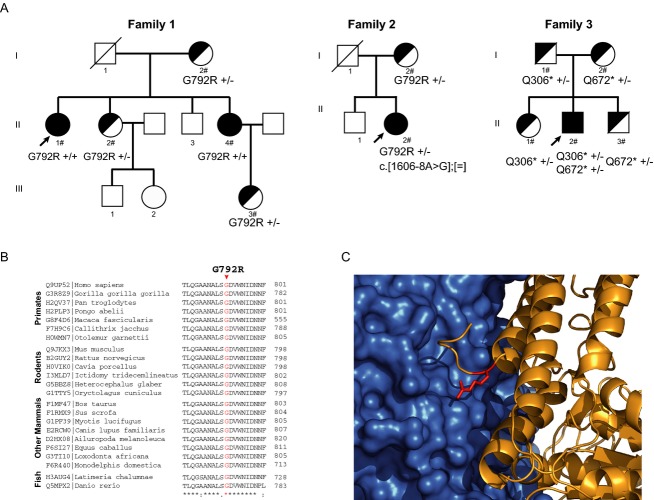
Hereditary hemochromatosis type 3 families: families and mutations in *TFR2* gene. (A) Pedigrees of three HH type 3 families. The probands are indicated with an arrow. Black symbols denote affected individuals, half-filled black symbols denote unaffected carriers. Individuals studied at the molecular level are indicated with the symbol #. (B) Partial amino acid sequence alignment of TFR2 protein in 23 species in the vicinity of p.Gly792Arg mutation. Uniprot accession numbers are reported for each sequence. Below the alignment a star (*) indicates 100% conservation of the amino acid, semicolons and dots indicate amino acids with similar but not identical properties. (C) Homology model of TFR2 dimerization interface. In blue, surface representation of a wild-type TFR2 monomer. In yellow, a cartoon representation of a TFR2 monomer with the p.Gly792Arg mutation colored in red. Note that G792R=Gly792Arg, Q306*=Gln306* and Q672*=Gln672*.

Proband II.1 (Fig.1A) is a Spanish woman that in September 1985 at 31 years of age presented with a history of 2 years of duration of hot flashes, asthenia and amenorrhea. Clinical examination revealed loss of body hair, low body weight, and slight affectation of small joints in the hands. She was diagnosed of hypogonadotropic hypogonadism (with normal Computed Tomography scan) and she began substitutive treatment. Additional laboratory tests showed a marked elevation of serum iron, serum ferritin and % transferrin saturation and low levels of hepcidin (Table1). A liver biopsy was performed showing a massive iron deposition in the liver parenchyma with incipient cirrhotic changes. With the diagnosis of HH, she began regular phlebotomy treatment, requiring up to 46 procedures to achieve a normal ferritin level (August 1993). The estimate total iron removed was 8.28 g. To maintain normal serum ferritin levels, she requires additional regular phlebotomies (48 were performed until June 2013, with an additional estimated iron removal of 8.64 g). Despite achievement of normal serum ferritin, we rarely obtain a normalization of serum iron or transferrin saturation. We ruled out the presence of mutations in *HFE* gene (Sanchez et al. 2000) and in the *HFE2, HAMP,* and *SLC40A1* genes. During these years, arthropathy continued to progress in her hands with severe deformation and disabling patient from work. The patient developed osteoporosis secondary to hypogonadism and was treated first with hormonal substitutes and thereafter with alendronate, calcium and vitamin D. The latest liver ecography performed showed no alteration in its structure and correct liver function. Her paternal and maternal ancestors were from an isolated mountain valley in the north of Spain. Her maternal grandmother died at 81 years from a liver cancer. In 1986 her younger sister was diagnosed with non-HFE HH presenting diabetes mellitus type I and hypersideremia.

**Table 1:** Main biochemical and genetic features of the TFR2-related hemochromatosis patients

Year of analysis	Reference values	Family 1, proband: II.1				Family 1, sister: II.4				Family 2, proband: II.2				Family 3, proband: II.2		
		1985	2003^1^	2007^1^	2013^1^	1992	1995	2005	2011	2007	2009^1^	2011^1^	2013^1^	2012	2013^1^	2014^1^
Serumiron (*μ*g/dL)	A: 59–158 P: 26–110	**232**	**287**	**278**	**222**	**200**	**198**	**228**	134	**236**	**326**	**278**	**325**	**217**	77	37
Serumferritin (ng/mL)	A:22–322 P: 23–70	**1950**	120	94	127			148	24	**3944**	82	176	236	**435**	**142**	19
Transferrinsaturation (%)	A: 20–50 P: 11–36	**100**		**93**	**65**			**100**	**53**	**96**		**84**	**89**	**96**	26	13
Hemoglobin (g/dL)	A: 135–175 P: 127–152	125		121	124		132	133	113	133	126	138	126	137	128	141
Hepcidin (ng/mL)^2^	A: 17–286 P: 16.58–74.57	n.d.	n.d.	n.d.	**9.1**	n.d.	n.d.	n.d.	**3.4**	n.d.	n.d.	n.d.	**6.3**	n.d.	n.d.	**13.42**
ALT (U/L)	A: 5–38 P: 5–32	22		25	27		21	22	30	**119**	11	18	15	21		
AST (U/L)	A: 5–41 P: 9–24	27		16	18		16	17	37	**212**	10	19	12	27		
Anti-HCV			Negative			Negative				Negative				Negative		
HBsAg			Negative			Negative				Negative				Negative		
TFR2 mutations (HGVS)																
Genbank mRNA: NM_003277.3		c.[2374 G>A];[2374 G>A]				c.[2374 G>A];[2374 G>A]				c.[1606-8A>G];[2374 G>A]				c.[916C>T];[2014C>T]		
Genbank protein: NP_003218.2		p.Gly792Arg]; p.Gly792Arg				p.Gly792Arg; p.Gly792Arg				Splicing defect; p.Gly792Arg				p.[(Gln306^*^)];[(Gln672^*^)]		

ALT, alanine transaminase; n.d., not determined; AST, aspartate transaminase; HCV, hepatitis C virus; HBsAg, surface antigen of the hepatitis B virus; HGVS, Human Genome Variation Society recommended mutation description. Nucleotide numbering uses +1 as the A of the ATG translation initiation codon in the reference sequence, with the initiation codon as codon 1. Reference biochemical values are according to Brugnara (2009) and Yee et al. (2009).

1 Indicates that the biochemical values were obtained after phlebotomy therapy. Values in bold are abnormal values above or below the reference value. Reference values: A, adult; P, pediatric values for a 14 year old boy.

2 Adult (A) serum hepcidin reference values according to (Ganz et al. 2008) are: mean of 121 ng/mL [5–95% CI: 29–254 ng/mL] for men and mean of 87 ng/mL [5–95% CI: 17–286 ng/mL] for women. Paediatric (P) serum hepcidin reference values using the serum hepcidin-25 c-ELISA kit from DRG in control boys (aged: 10–12 years old) according to (Sdogou et al. 2014) are 16.58–74.57 ng/mL (range).

#### Family 2

The second patient is a 23-year-old Spanish woman who came to our attention in September 2007 for an elevation of serum ferritin (Fig.1A). She also referred an alteration in hepatic enzymes at 15 years old of age. The liver transaminases (Aspartate transaminase (AST) and Alanine transaminase (ALT)), serum ferritin, serum iron, transferrin saturation and hepcidin values and evolution are listed in Table1. Hepatitis B and C infections were excluded and a *HFE* H63D homozygous variation was identified. No further genetic changes were documented in the *HFE*, *HFE2*, *HAMP*, or *SLC40A1* genes. There was no family history of iron disorders, her father died due to a hepatic viral hepatopathy. Liver biopsy revealed massive iron deposition in the liver parenchyma with moderate fibrosis. The hepatic iron concentration measured by quantitative Magnetic Resonance Imaging was of 267 *μ*mol/g dry weight (normal reference value <36 *μ*mol/g). The echocardiographic study revealed a normal cardiac ejection fraction and no valvular lesions. She began weekly phlebotomy with administration of erythropoietin because of poor hematopoietic recovery. A total of 9 g of iron was removed until normalization of ferritin (March 2009), and three additional grams of iron were removed afterwards. The patient is now in good condition.

#### Family 3

The third patient is a young boy of 14 years old, diagnosed at 12 years old because of recurrent dizziness (Fig.1A). The neurological and cardiologic examination showed no abnormalities; however, laboratory tests showed an increase in transferrin saturation and hyperferritinemia (see Table1). There was no family history of iron disorders or consanguinity. The presence of C282Y and H63D variations in *HFE* gene were ruled out and complete sequencing of *HFE2* and *HAMP* genes revealed no pathological mutations. The liver iron concentration measured by quantitative magnetic resonance was increased (180 *μ*mol/g) but there was no evidence of myocardial iron overload (T2*: 44 msec).The endocrinological analysis and echocardiogram were normal. He began bi-weekly treatment with phlebotomy (300 mL) with good clinical tolerance. Ferritin became normal after removing 2.4 g of iron with a total of 16 phlebotomies.

Written informed consent for molecular genetic analyses was obtained from the probands and relatives of the three families according to the guidelines of our institution and the study protocol conforms to the ethical guidelines of the 2002 Helsinki declaration. All procedures were done according to the manufacturer's instructions, unless stated otherwise.

### Hepcidin assay

Hepcidin concentrations in patient's plasma/serum samples were quantified by competition enzyme-linked immunoassay (C-ELISA) using the hepcidin-25 (human) enzyme immunoassay kit (Bachem, Torrance, CA, USA) according to the manufacturer's protocol. Samples and standards were run in duplicate. Plasma samples were diluted 1 in 10 or 1 in 50 in supplied standard diluent (peptide-cleared human serum) and analyzed using a 10-point twofold serial dilution (maximum concentration, 25 ng/mL) standard curve. Hepcidin concentrations were interpolated from standard curves generated by a four-parameter logistic nonlinear regression model using Prism (GraphPad Software Inc., La Jolla, CA). Appropriate dilutions were used to obtain readings inside the linear region of the curve.

### Oligonucleotides

All oligonucleotides sequences are listed in Table S2.

### DNA extraction, PCR amplification, and sequencing

DNA was extracted from peripheral blood using the FlexiGene DNA kit or QIAamp-DNA-Blood-Mini kit (Qiagen, CA, USA). PCR amplifications were done with 50 ng of genomic DNA.PCR conditions are available upon request. The PCR product was processed with exonuclease I and antarctic phosphatase (New England Biolabs, MA, USA), and sequenced. Sequencing results were analyzed using Mutation Surveyor (SoftGenetics LLC, PA) or Chromas Lite 2.01 (Technelysium Pty Ltd, South Brisbane, Australia) softwares. All sequencing was done by conventional Sanger method at the GATC BIOTECH Company (Constance, Germany).

Mutant alleles are named according to journal guidelines (www.hgvs.org) and have been submitted to the Leiden Open Variation Database (http://www.lovd.nl/TFR2).

### Plasmids and mutagenesis

Plasmids pcDNA3.1(+) and pCMV-Tag4-expressing TFR2 protein tagged with N-terminus and C-terminus FLAG protein (Einhauer and Jungbauer 2001) respectively were a kind gift from Dr Clara Camaschella. Site-directed mutagenesis for p.Gly792Arg was done in these plasmids by a standard protocol with primers G792R-f and G792R-r.

The splicing reporter minigene plasmid pCMV-DiSophie was a kind gift from Dr Juan Valcarcel. TFR2 genomic region containing exons 13, 14, and 15 and intronic regions (size 423 pb) was cloned into this plasmid to obtain TFR2-DiSophie plasmid for splicing studies. Briefly, first PCR was done on genomic DNA from a healthy control followed by a second PCR to incorporate PT1 and PT2 unique sequences (Sakamoto et al. 1992) with *Kpn*1 and *Not*1 restriction sites. This second PCR product was purified using GFX™ PCR DNA and gel band purification kit (GE Healthcare Ltd, Little Chalfont, UK), digested and cloned. The c.1606-8A>G variant was introduced into TFR2-DiSophie by site-directed mutagenesis using primers c.1606-8A>G-f and c.1606-8A>G-r (Table S2).

### Cells and cell lines

Huh7 and HeLa cells were kind gifts from Matthias W. Hentze, EMBL, Heidelberg, Germany. Cells were grown in Dulbecco's modified Eagle's medium (Labclinics, S.A., Spain) supplemented with 10% fetal bovine serum (FBS), 2 mmol/L l-glutamine, 1× antibiotic-antimycotic (penicillin, streptomycin, amphotericin), 1 mmol/L sodium pyruvate.

Human peripheral blood mononuclear cells (PBMCs) were extracted from fresh samples of probands and controls by Ficoll (Rafer, SL, Spain) density gradient, and grown in RPMI medium with FBS, l-glutamine, antibiotics and 0.1 mg/mL phytohemagglutinin for 72 h. Puromycin (50 *μ*g/mL) (Sigma-Aldrich, St. Louis, MO, USA) was added 5 h before RNA extraction.

### Immunofluorescence

Cells were grown to a confluency of 50–80% in millicell EZ 8-well slides (Millipore, Iberica, Madrid, Spain) and transfected with wild-type or p.Gly792Arg mutated TFR2-FLAG constructs by Genejuice (Merck Chemicals, Ltd, NJ, USA). At 48 h posttransfection, cells were fixed with 4% paraformaldehyde (Sigma Aldrich), permeabilized or not with Triton X-100 0.1%; incubated with the appropriate antibodies (mouse monoclonal anti-FLAG, [Sigma], rat monoclonal anti-E-cadherin [Millipore, CA, USA] or rabbit polyclonal anti-GRP78 BiP [Abcam, Cambridge, UK] as primary; AlexaFluor 488 rabbit-anti-rat, AlexaFluor 488 goat-anti-rabbit and AlexaFluor 568 goat-anti-mouse [Invitrogen Molecular Probes, OR, USA] as secondary); washed with PBS-Tween 0.02% and PBS and mounted in DAPI-containing mounting medium (Vector Laboratories, CA, USA). Epifluorescence images were acquired using a Leica DMI 6000B microscope at ×63 objective and analyzed by ImageJ software (Bethesda, MD).

### Splicing studies

For c.1606-8A>G minigene studies, HeLa cells were cultured in 100 mm plates (Nunc; Thermofisher Scientific, MA, USA) to a confluency of 50–80% and transfected by Genejuice with wild-type or c.1606-8A>G TFR2-DiSophie constructs. At 24 h posttransfection, total RNA was extracted with TRIzol (Ambion Life Technologies, NY, USA). Two *μ*g of total RNA was DNAse treated (Promega, WI) and reverse transcribed using GoScript™ kit (Promega, WI, USA). About 100 ng cDNA was used for RT-PCR with PT1-sense and exon 14-antisense primers (Table S2). For studies in family 2 patient II.2, total RNA was extracted from patient's PBMCs, and processed as described above using primers TfR2-II.2-F and TfR2-II.2-R. RT-PCR products were run on a 2% or 3% agarose gel, cloned into pCR®-Blunt or pCR®-2.1 plasmids from Invitrogen Life Technologies, NY, USA Zero-Blunt® or TA-cloning® kits and sequenced.

### Bioinformatics and computational studies

SIFT (Kumar et al. 2009) and PolyPhen-2 programmes (Adzhubei et al. 2010) were used to study the functional effects of p.Gly792Arg mutation. The Human Splice Finder program (Desmet et al. 2009) (http://www.umd.be/HSF/) was used to analyze the putative splicing effects of the TFR2 c.1606-8A>G intronic variant.

TFR2 multiple sequence alignment was built following a two-step procedure. First, human TFR2 was used to query the protein sequence database UniRef100 (Suzek et al. 2007) with Psi-blast (version 2.2.28+, number of iterations set to 2, and -e to 0.001) (Altschul et al. 1997). From the resulting list of candidates, we discarded those that were less than 40% identical to human TFR2. Second, the sequences of the final candidates were aligned with the program MUSCLE (Edgar 2004; version 3.8.13, default parameters).

A structural model of the TFR2 dimer was created using the experimental structure of TFR1 (PDB: 1DE4; 48% sequence identity) and version 9.8 of the standard modeling package Modeler. Relative accessibility at the mutation site was obtained running the program NACCESS (Hubbard et al. 1991; version 2.1.1, default parameters).

## Results

As described in the patients and methods section all affected patients presented signs and/or symptoms of iron overload including high serum iron, high serum ferritin, and high transferrin saturation levels, low hepcidin levels, liver iron overload, diabetes mellitus, cirrhosis or fibrosis, and other associated complications typically of HH (hypogonadotropic hypogonadism, arthropathy) at an early age (Table1).

In the adult patients, iron removal by phlebotomies was very high (16.92 and 12 g, respectively, in both probands) and confirmed the HH diagnosis.

The proband of family 2 was homozygous for the *HFE* H63D variation. However, this genotype is not associated with the HH pathogenicity, cannot explain the severe phenotype of the patient and its allele frequency in the Spanish population is one of the highest reported world-wide (European Association For The Study Of The Liver 2010; Sanchez et al. 2003).

**Figure 2:**
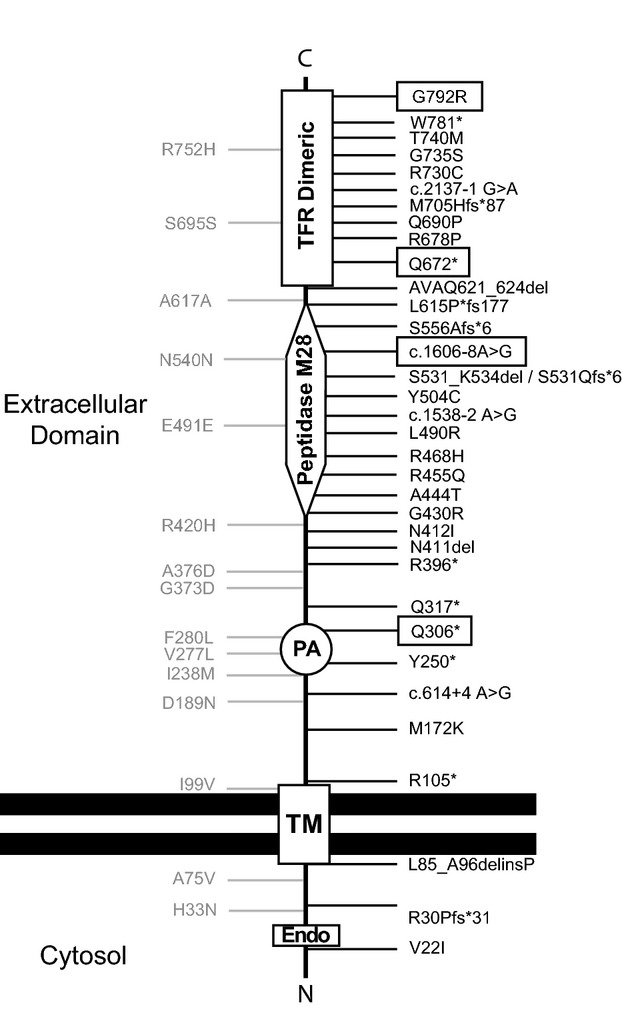
Schematic localization of literature reported and new TFR2 mutations. Pathogenic mutations are reported in black at the right side and unproven variations are reported in grey at the left site (see also Table S1). Mutations described in this work are boxed. Domains: Endo: endocytic signal; TM, transmembrane domain; PA, protease-associated domain; Peptidase M28, TFR Dimeric domain. For space concerns we report here the *TFR2* protein changes using the one-letter amino acid code.

Genetic analyses of *TFR2* revealed that the affected members of families 1 and 2 present a previously described (Lee and Barton 2006) but uncharacterized missense mutation, p.Gly792Arg. The proband II.1 and her sister II.4 in family 1 present this mutation in homozygous state while the proband II in family 2 presents this mutation in compound heterozygous state with a novel splicing mutation (c.1606-8A>G) (Figs.1A, 2). To the best of our knowledge, this is the first time that the p.Gly792Arg mutation is reported in a pedigree (family 1) in a homozygous state.

We detected two novel *TFR2* nonsense mutations (Gln306* and Gln672*) in compound heterozygous state in proband II.2 of family 3, a pediatric case. These mutations are absent from public databases (ENSEMBL, NCBI, 1000 Genomes) and each one was inherited from a heterozygous and unaffected parent. The proband's siblings were also carrying only one single *TFR2* mutation (Fig.1A). In the proband, these two mutations will produce a truncated no-functional TFR2 protein lacking the transferrin receptor dimeric domain from both alleles (Fig.2).

### Bioinformatics and computational studies on the p.Gly792Arg mutation

The mutation p.Gly792Argis located in at the carboxi-terminal end of the TFR2 protein inside the transferrin receptor dimeric domain (Fig.2) and was predicted to be deleterious by SIFT(Kumar et al. 2009) and PolyPhen-2 (Adzhubei et al. 2010) programs. To confirm and understand its disruptive nature we performed a progressive series of bioinformatics and computational analyses. Study of the multiple sequence alignment of the TFR2 family (23 sequences, Fig.1B) showed that glycine at position 792 is absolutely conserved, pointing to its relevance to protein function/structure. Mutations breaking such extreme conservation patterns are generally associated with severe functional loss (Ferrer-Costa et al. 2002), particularly when involving large deviations in physico-chemical properties, as is the case for p.Gly792Arg, for which both volume (100 A^3^) and hydrophobicity (1.37 Kcal/mol) changes are drastic.

To refine our analyses, we modeled the structure of TFR2 based on TFR1 PDB structure 1DE4 (48% sequence identity with TFR2). The structure model shows that the G792 mutation is located at the end of an alpha-helix, near TFR2's C-terminus and within its dimerization domain. Further structural analysis shows that this location is half-buried (37.6% relative accessibility, measured with NACCESS (Hubbard et al. 1991)) and at the interphase between TFR2 monomers (Fig.1C). This result, in accordance with the previous bioinformatics analyses, supports the idea that the impact of p.Gly792Arg mutation on TFR2 function is the result of a combined negative effect both on dimer structure and monomer stability.

### p.Gly792Arg mutation impairs TFR2 localization to the plasma membrane

**Figure 3:**
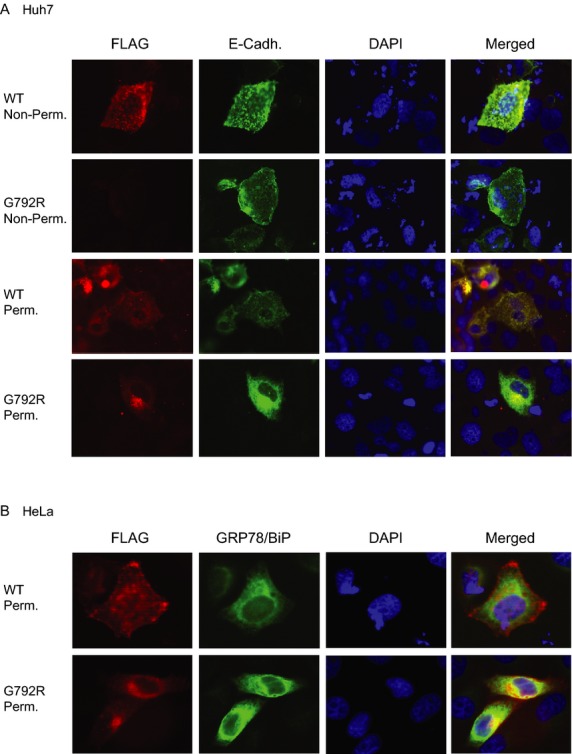
Immunofluorescence studies of the *TFR2* p.Gly792Arg mutant in human cell lines. (A) C-terminal FLAG wild-type or mutated p.Gly792Arg TFR2 constructs were transiently transfected and visualized using anti-FLAG antibody (red) in an epifluorescence microscope in permeabilized and nonper-meabilized Huh7cells. E-Cadherin was used as a membrane protein control (green). DAPI (blue) was used to detected DNA and visualize nuclear morphology. (B) N-terminal FLAG wild-type or mutated p.Gly792Arg TFR2 constructs were transiently transfected as above and visualized in permeabilized HeLa cells, using anti-FLAG (red) and anti-GRP78-BiP (green, ER marker) antibodies. Note that G792R=Gly792Arg.

In order to assess the effect of p.Gly792Arg mutation on TFR2 intracellular localization, we transiently transfected two independent cell lines (Huh7 and HeLa cells) with wild-type or mutated (p.Gly792Arg) TFR2-FLAG chimeric constructs and assessed TFR2 localization by immunofluorescence. Contrary to wild-type FLAG-TFR2 protein, the p.Gly792Arg mutant protein is not detected at the membrane surface and it is intracellularly retained (Fig.3A and B), as indicated by the absence of staining detection in nonpermeabilized HUH7 cells when using a C-terminal TFR2-FLAG construct (Fig.3A panel 2) or by non-co-localization with the plasma membrane control protein E-Cadherin in permeabilized cells (using both C-terminal, Fig.3A and, N-terminal, data not shown, TFR2-FLAG construct). The mutant protein partially overlaps with the endoplasmic-reticulum marker GRP78-BiP (Fig.3B). Overall, these results indicate that the plasma membrane trafficking of the mutated p.Gly792Arg TFR2 protein is impaired.

### Effect of the c.1606-8A>G variant in TFR2 splicing

Patient II.2 from family 2 presents a novel intronic variation c.1606-8A>G (intron 13–14) in compound heterozygous state together with the p.Gly792Arg mutation. This intronic change was absent in the mother and we infer that was inherited from the deceased father or alternatively acquired as a de novo mutation. This variant is not described in any databases (ENSEMBL, NCBI, 1000 Genomes). The Human Splice Finder splicing program predicted that the substitution of an A by a G at position -8 of intron 13-14 of TFR2 would create a new acceptor splicing site (aa>ag) that may interfere with TFR2 splicing.

**Figure 4:**
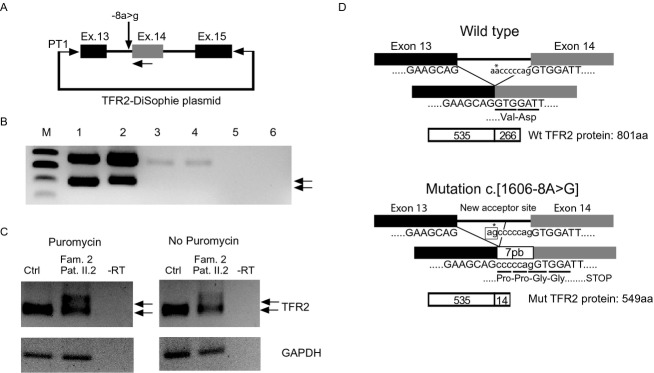
Splicing studies for c.1606-8A>G mutation. (A) Schematic representation of the minigene TFR2-DiSophie construct including TFR2 genomic region comprising exon 13 to exon 15. Boxes represent exons and lines introns or vector sequence. Horizontal arrows denotes PT1 and exon 14 primer used for amplification. (B) Analysis of the splicing pattern of TFR2-DiSophie construct by RT-PCR. M: DNA marker, lane 1: wild-type plasmid, lane 2: c.1606-8A>G mutated plasmid, lane 3 and 4: no transfection reagent controls, lane 5 and 6: negative RT-PCR controls. Arrows indicate the TFR2 mRNA-specific splicing amplifications excluding intron 13–14. Notice the band in lane 2 (c.1606-8A>G mutated plasmid) is slightly shifted up. (C) RT-PCR analysis in RNA from PBMCs of family 2 patient II.2 (heterozygous for the c.1606-8A>G mutation) in the presence or absence of puromycin. GAPDH was used as RT-PCR control. (D) Schematic representation of the effect of the c.1606-8A>G mutation on TFR2 mRNA and protein. Black and gray boxes represent exons 13 and 14 and a horizontal line the intron. Open boxes represent the TFR2 protein. The -8A>G substitution is indicated with an asterisk (*), the new acceptor site is boxed. Capital letters denotes coding nucleotides and lowercase letters denotes intronic sequences. Codons are underlined and the encoded amino acid is shown below. The c.1606-8A>G splicing mutation leads to the inclusion of seven intronic nucleotides (cccccag) also indicated as 7 bp.

To address the possible splicing implications of this variation, we transiently transfected HeLa cells with a minigene construct (Fig.4A) containing the wild-type or mutant c.1606-8A>G TFR2 genomic region between exons 13 and 15.

RNA was isolated and analyzed by RT-PCR as described previously (Sakamoto et al. 1992) using primers corresponding to the transcribed vector sequences (PT1) and exon 14 of TFR2 (Fig.4A), thus allowing specific detection of transcripts derived from the TFR2 minigene and not from endogenous TFR2 transcripts. A slightly shifted band was detected in the RT-PCR from the mutated c.1606-8A>G TFR2 construct compared to the wild-type (Fig.4B, arrows, lane 2 vs. lane 1). Sequencing of the wild-type and mutated bands revealed that the c.1606-8A>G TFR2 RT-PCR band contains an insertion of seven nucleotides (cccccag) from intron 13–14 of TFR2 and confirm the predicted recognition of a new splicing acceptor site derived from the A>G substitution (Fig.4D). The aberrant mRNA is predicted to produce a truncated TFR2 protein (549 amino acids vs. 801 amino acids of the wild-type TFR2 protein) with the inclusion of 14 extra amino acids followed by a premature stop codon (Fig.4D).

RT-PCR analysis on RNA extracted from the patient's PBMCs treated with puromycin (a translation inhibitor that suppresses nonsense-mediated decay machinery) confirmed that in vivo there was the same seven intronic nucleotides (cccccag) insertion as reported with the minigene method (Fig.4C). In untreated PBMCs we could still detect the *TFR2* pathogenic allele suggesting that the aberrant TFR2 mRNA is not completely subjected to degradation by the nonsense mediated decay machinery (Fig.4D) and therefore, the mechanism causing TFR2 deficiency of this allele comprises RNA and protein degradation.

Hence, we confirm with two independent strategies, in vitro (with a minigene construct) and in vivo (patient's PBMCs), that the c.1606-8A>G variation is a pathogenic splicing mutation that creates a novel splicing acceptor site and generates an aberrant mRNA not fully subjected to mRNA degradation by the nonsense-mediated decay system.

## Discussion

In the current work, we describe three novel TFR2 mutations (two nonsense and one splicing mutation) and functionally characterize one previously described missense mutation and the new splicing variant. The p.Gly792Arg missense mutation was previously reported in an HH patient in a compound heterozygous state with 2 other *TFR2* mutations: Arg396* and Arg455Gln (Lee and Barton 2006). In that patient, the p.Gly792Arg variant was detected in cis with the nonsense mutation Arg396*. Therefore, the authors conclude that the functional effect of TFR2 p.Gly792Arg was unknown, and in the context of their reported patient was irrelevant. In addition, this missense mutation (p.Gly792Arg) was also recently been reported *in trans* and in heterozygous state in two patients; one bearing the p.Met705Hisfs*87 mutation and another patient with the variation p.Ala444Thr in *TFR2* gene together with the ferroportin mutation p.Gly204Ser (Bardou-Jacquet et al. 2013). Here, we reported for the first time a HH type 3 family homozygous for this variation and demonstrated by bioinformatics, computational and coimmunofluorescence studies that indeed this is a pathogenic mutation that affects plasma membrane TFR2 localization in human cell lines (Huh7 and HeLa). We conclude that most probably this intracellular retention will subsequently impair TFR2 downstream signaling of hepcidin production and will lead to Hemochromatosis type 3 disease.

A second HH type 3 patient (patient II.2 from family 2) was compound heterozygous for this same mutation (p.Gly792Arg) and for a novel intronic variation (c.1606-8A>G). To date only three splicing mutations have been proven to be pathogenic in patients with HH type 3: c.1538-2A>G in compound heterozygous state with R396* mutation (Gerolami et al. 2008), c.2137-1G>A and c.614+4A>G both in homozygous state (Biasiotto et al. 2008; Pelucchi et al. 2009; Radio et al. 2014). Here, we demonstrate that the c.1606-8A>G variation leads to the recognition of a new acceptor splicing site, that produce an aberrant TFR2 mRNA and protein. Therefore, other putative splicing defects in *TFR2* gene outside the canonical donor and acceptor sites should be taken into consideration and functionally tested to investigate if they are producing a pathologic allele.

Our studies confirm that HH type 3 patients present with severe disease phenotypes at a younger age compared with HFE-related HH as suggested in other studies (Pietrangelo et al. 2005). Therefore, *TFR2* genotyping should be considered in suspected hemochromatosis patients negative for *HFE* mutations and with early-onset of iron overload. In addition, it should be performed in juvenile HH forms without mutations in the *HAMP* or *HFE2* genes. The genetic test allows diagnostic confirmation and early implementation of therapy avoiding future severe clinical manifestations.

## Supporting Information

**Table S1.** TRF2 mutations reported in the literature and in this work. **Table S2.** Cloning and sequencing oligonucleotides used in this study.
