# Supplementary material for: Functional consequences of transferrin receptor-2 mutations causing hereditary hemochromatosis type 3
Source: Mol Genet Genomic Med. 2015 Mar 6;3(3):221–32. doi: 10.1002/mgg3.136 (PMC4444164; doi:10.1002/mgg3.136)
Supplement: Supplementary file 1 — Table S1. TRF2 mutations reported in the literature and in this work. Table S2. Cloning and sequencing oligonucleotides used in this study. [file mgg30003-0221-sd1.docx]

**Supplementary data for manuscript:**

**Functional consequences of transferrin receptor-2 mutations causing Hereditary Haemochromatosis type 3**

Ricky Joshi^1*^, Maya Shvartsman^1*^, Erica Morán^1^, Sergi Lois^2^, Jessica Aranda^1^, Anna Barqué^1^, Xavier de la Cruz^2^, Miquel Bruguera^3^, José Manuel Vagace^5^, Guillermo Gervasini^6^, Cristina Sanz^4^, Mayka Sánchez^1,7^

**Supplementary Table 1.** TRF2 mutations reported in the literature and in this work

**Supplementary References**

**Supplementary Table 2.** Cloning and sequencing oligonucleotides used in the study.

**Supplementary Table 1. Pathogenic TRF2 mutations reported in the literature* and in this work**

| **Ancestry** | **Nº Families** | **Nº Affected patients** | **Molecular defects. TFR2 protein: NP_003218.2. TFR2 gene: NM_003227.3** | | | | **Clinical significance** | | **rs ID (NCBI SNP)** | **Reference** |
| --- | --- | --- | --- | --- | --- | --- | --- | --- | --- | --- |
|  |  |  | **Mutation 1** | **Domain mutation 1** | **Mutation 2** | **Domain mutation 2** | **Mutation 1** | **Mutation 2** |  |  |
| Iranian | 1 | 1 | AVAQ 621_624del (reported as AVAQ 594_597del) (c.1861_1872del) | Extracellular | AVAQ 621_624del (reported as AVAQ 594_597del) (c.1861_1872del) | Extracellular | Pathogenic | Pathogenic | rs80338888 | [1] |
| Spanish | 1 | 1 | p.G373D (c.1118G>A) | Extracellular | Not found | - | NA | - | rs202221581, MAF: < 0.01 | [2] |
| Spanish | 1 | 1 | p.R420H (c.1259G>A) | Extracellular | Not found | - | NA | - | rs146901123, MAF: < 0.01 | [3] |
| Italian | 1 | 1 | c.614+4A>G | Intronic | c.614+4A>G | Intronic | Pathogenic | Pathogenic | - | [4] |
|  | 1 | 1 | p.F280L (c.840C>G) | PA | Not found | - | NA | - | rs151198873, MAF: < 0.01 | [5] |
| Italian | 1 | 1 | p.R396* (c.1186C>T) | Extracellular | c.1538-2 A>G | Intronic | Pathogenic | Pathogenic | R396*: rs80338882; 1538-2 A>G: rs371347559 | [6] |
| Italian | 1 | 1 | p.N411del (c.1231_1233delAAC) | Extracellular | p.A444T (c.1330G>A) | Peptidase M28 | Pathogenic | Pathogenic | N411del: rs80338883; A444T: rs80338884 | [7] |
| Italian | 1 | 2 | c.2137-1G>A (reported as IVS17+5636G>A) | Intronic | c.2137-1G>A (reported as IVS17+5636G>A) | Intronic | Pathogenic | Pathogenic | rs80338890 | [7] |
| Native American/white | 1 | 1 | p.D189N (c.565G>A) | Extracellular | Not found in TFR2, HAMP promoter mutation -443C>T in heterozygocity | - | NA | - | rs144967912, MAF: NA | [8] |
| Taiwanese | 1 | 1 | p.R468H (reported as p.R481H) (c.1403G>A ) | Peptidase M28 | Not found | - | Pathogenic | - | rs80338885 | [9] |
| Italian | 1 | 2 | p.M172K (c.515T>A) | Extracellular | p.M172K (c.515T>A) | Extracellular | Pathogenic | Pathogenic | rs80338879 | [10] |
| Scoth-Irish American | 1 | 1 | p.R396* (c.1186C>T) in *cis* with p.G792R (c.2374G>A) | R396*: Extracellular; G792R: TRF Dimeric | p.R455Q (c.1364G>A) | Peptidase M28 | R396*: Pathogenic; G792R: not known | Pathogenic | R369*: rs80338882; R455Q: rs41303501 | [11] |
| Italian | 1 | 3 | p.Q317* (c.949C>T) | Extracellular | p.Q317* (c.949C>T) | Extracellular | Pathogenic | Pathogenic | rs80338881 | [12] |
| Japanese | 1 | 1 | p.S556Afs*6 (reported as p.V561*) (c.1665delC) | Peptidase M28 | p.S556Afs*6 (reported as p.V561*) (c.1665delC) | Peptidase M28 | Pathogenic | Pathogenic | rs80338887 | [13] |
| Japanese | 1 | 1 | p.L490R (c.1469T>G) | Peptidase M28 | p.L490R (c.1469T>G) | Peptidase M28 | Pathogenic | Pathogenic | rs80338886 | [13] |
| North French | 1 | 2 | p.R105* (c.313C>T) | Near TM | p.R105* (c.313C>T) | Near TM | Pathogenic | Pathogenic | rs80338878 | [14] |
| Italian | 2 | 2 | p.Y250* (c.750C>G) | PA | p.Y250* (c.750C>G) | PA | Pathogenic | Pathogenic | rs80338880 | [15] |
| Japanese | 1 | 3 | AVAQ 621_624del (reported as AVAQ 594_597del) (c.1861_1872del) | Extracellular | AVAQ 621_624del (reported as AVAQ 594_597del) (c.1861_1872del) | Extracellular | Pathogenic | Pathogenic | rs80338888 | [16] |
| Italian | 3 | 3 | p.V22I (c.64G>A) | Near Endo | Not found in TFR2 (1 patient also homozygous for HFE C282Y) | - | Pathogenic | - | V22I: rs80338876 | [17] |
| Italian | 1 | 3 | AVAQ 621_624del (reported as AVAQ 594_597del) (c.1861_1872del) | Extracellular | AVAQ 621_624del (reported as AVAQ 594_597del) (c.1861_1872del) | Extracellular | Pathogenic | Pathogenic | rs80338888 | [18] |
| Portuguese | 1 | 3 | p.Q690P (c.2069A>C) | TFR Dimeric | p.Q690P (c.2069A>C) | TFR Dimeric | Pathogenic | Pathogenic | rs80338889 | [19] |
| Asiatic | 1 | 1 | p.R455Q (c.1391G>A) | Peptidase M28 | not found in TFR2 (patient also homozygous for HFE C282Y) | - | Pathogenic | - | R455Q: rs41303501 | [20] |
| Italian | 1 | 6 | p.R30Pfs*31 (reported as p.E60X) (c.88dup, reported as c.84_88insC) | Cytosolic | p.R30Pfs*31 (reported as p.E60X) (c.88dup, reported as c.84_88insC) | Cytosolic | Pathogenic | Pathogenic | rs80338877 | [21] |
| Italian | 1 | 1 | p.M172K (c.515T>A) | Extracellular | p.M172K (c.515T>A) | Extracellular | Pathogenic | Pathogenic | rs80338879 | [21] |
| Italian | 2 | 6 | p.Y250* (c.750C>G) | PA | p.Y250* (c.750C>G) | PA | Pathogenic | Pathogenic | rs80338880 | [22] |
| Spanish | 1 | 1 | p.F280L (c.840C>G) | PA | not found in TFR2 (patient also heterozygous for HFE H63D) | - | NA | - | rs151198873, MAF: < 0.01 | [23] |
| African | 1 | 1 | p.G430R (c.1288G>A) | Peptidase M28 | p.Y504C (c.1511A>G) | Peptidase M28 | Pathogenic | Pathogenic | n.d.; n.d. | [24] |
| Italian | 1 | 1 | p.T740M (c.2219C>T) | TFR Dimeric | p.T740M (C.2219C>T) | TFR Dimeric | Pathogenic | Pathogenic | rs150806077, MAF: < 0.01 | [25] |
| Italian | 1 | 1 | p.L615Pfs*177 (c.1841_1842insG) | Near Peptidase M28 | Not found | - | Pathogenic | - | n.d. | [25] |
| Italian | 1 | 1 | p.S531_K534del / p.S531Qfs*6 (c.1591_1595delinsCAGGCAAGAGCAC) | Peptidase M28 | Not found | - | Pathogenic | - | n.d. | [25] |
| French | 1 | 1 | p.R678P (c.2033G>C) | TFR Dimeric | p.R678P (c.2033G>C) | TFR Dimeric | Pathogenic | Pathogenic | n.d. | [26] |
| French | 1 | 1 | p.N412I (c. 1235A>T) | Extracellular | p.N412I (c. 1235A>T) | Extracellular | Pathogenic | Pathogenic | n.d. | [26] |
| North Africa | 1 | 1 | p.G430R (c.1288G>A) | Peptidase M28 | p.G430R (c.1288G>A) | Extracellular | Pathogenic | Pathogenic | n.d. | [26] |
| French | 1 | 1 | p.L85_A96delinsP (c.254_286 +9del) | TM | p.G735S (c.2203G>A) | TFR Dimeric | Pathogenic | Pathogenic | n.d.; n.d. | [26] |
| French | 1 | 1 | p.A444T (c.1330G>A) | Peptidase M28 | p.G792R (c.2374G>A) | TFR Dimeric | Pathogenic | Pathogenic | A444T: rs80338884; G792R: rs80338891 | [26] |
| French | 1 | 1 | p.R730C (c.2188C>T) | TFR Dimeric | p.W781* (c.2343G>A) | TFR Dimeric | Pathogenic | Pathogenic | n.d.; n.d. | [26] |
| French | 1 | 1 | p.M705Hfs*87 (c.2112dup) | TFR Dimeric | p.G792R (c.2374G>A) | TFR Dimeric | Pathogenic | Pathogenic | n.d.; rs80338891 | [26] |
| Spanish | 1 | 2 | p.G792R (c.2374G>A) | TFR Dimeric | p.G792R (c.2374G>A) | TFR Dimeric | Pathogenic | Pathogenic | rs80338891 | This work |
| Spanish | 1 | 1 | p.G792R (c.2374G>A) | TFR Dimeric | c.1606-8A>G | Intronic | Pathogenic | Pathogenic | rs80338891 / n.d. | This work |
| Spanish | 1 | 1 | p.Q306* (c.916C>T) | PA | p.Q672* (c.2014C>T) | TFR Dimeric | Pathogenic | Pathogenic | n.d.; n.d. | This work |

For space concerns we report here the *TFR2* protein changes using the one-letter amino acid code.

*We do not include in this table variations reported as non-pathogenic, polymorhpisms or rare synonymous substitutions not proven to be pathogenic such as the following TFR2 variants: H33N, A75V, I99V, I238M, V277L, A376D, E491E, N540N, A617A, S695S, R752H.

PA: protease associated domain. TM: transmembrane domain. N.d. not described. N. A. not available

**Supplementary References**

[1] Zamani F, Bagheri Z, Bayat M, Fereshtehnejad SM, Basi A, Najmabadi H, et al. Iranian hereditary hemochromatosis patients: baseline characteristics, laboratory data and gene mutations. Med Sci Monit 2012;18:CR622-629.

[2] Del-Castillo-Rueda A, Moreno-Carralero MI, Cuadrado-Grande N, Alvarez-Sala-Walther LA, Enriquez-de-Salamanca R, Mendez M, et al. Mutations in the HFE, TFR2, and SLC40A1 genes in patients with hemochromatosis. Gene 2012;508:15-20.

[3] Del Castillo-Rueda A, Moreno-Carralero MI, Cuadrado-Grande N, Mendez M, Moran-Jimenez MJ. [Hyperferritinemia, ferropenia and metabolic syndrome in a patient with a new mutation of gene TFR2 and another in gene FTL. A family study]. Med Clin (Barc) 2011;137:68-72.

[4] Pelucchi S, Mariani R, Trombini P, Coletti S, Pozzi M, Paolini V, et al. Expression of hepcidin and other iron-related genes in type 3 hemochromatosis due to a novel mutation in transferrin receptor-2. Haematologica 2009;94:276-279.

[5] Mendes AI, Ferro A, Martins R, Picanco I, Gomes S, Cerqueira R, et al. Non-classical hereditary hemochromatosis in Portugal: novel mutations identified in iron metabolism-related genes. Ann Hematol 2009;88:229-234.

[6] Gerolami V, Le Gac G, Mercier L, Nezri M, Berge-Lefranc JL, Ferec C. Early-onset haemochromatosis caused by a novel combination of TFR2 mutations (p.R396X/c.1538-2 A>G) in a woman of Italian descent. Haematologica 2008;93:e45-46.

[7] Biasiotto G, Camaschella C, Forni GL, Polotti A, Zecchina G, Arosio P. New TFR2 mutations in young Italian patients with hemochromatosis. Haematologica 2008;93:309-310.

[8] Barton JC, Acton RT, Leiendecker-Foster C, Lovato L, Adams PC, Eckfeldt JH, et al. Characteristics of participants with self-reported hemochromatosis or iron overload at HEIRS study initial screening. Am J Hematol 2008;83:126-132.

[9] Hsiao PJ, Tsai KB, Shin SJ, Wang CL, Lee ST, Lee JF, et al. A novel mutation of transferrin receptor 2 in a Taiwanese woman with type 3 hemochromatosis. J Hepatol 2007;47:303-306.

[10] Majore S, Milano F, Binni F, Stuppia L, Cerrone A, Tafuri A, et al. Homozygous p.M172K mutation of the TFR2 gene in an Italian family with type 3 hereditary hemochromatosis and early onset iron overload. Haematologica 2006;91:ECR33.

[11] Lee PL, Barton JC. Hemochromatosis and severe iron overload associated with compound heterozygosity for TFR2 R455Q and two novel mutations TFR2 R396X and G792R. Acta Haematol 2006;115:102-105.

[12] Pietrangelo A, Caleffi A, Henrion J, Ferrara F, Corradini E, Kulaksiz H, et al. Juvenile hemochromatosis associated with pathogenic mutations of adult hemochromatosis genes. Gastroenterology 2005;128:470-479.

[13] Koyama C, Wakusawa S, Hayashi H, Suzuki R, Yano M, Yoshioka K, et al. Two novel mutations, L490R and V561X, of the transferrin receptor 2 gene in Japanese patients with hemochromatosis. Haematologica 2005;90:302-307.

[14] Le Gac G, Mons F, Jacolot S, Scotet V, Ferec C, Frebourg T. Early onset hereditary hemochromatosis resulting from a novel TFR2 gene nonsense mutation (R105X) in two siblings of north French descent. Br J Haematol 2004;125:674-678.

[15] Piperno A, Roetto A, Mariani R, Pelucchi S, Corengia C, Daraio F, et al. Homozygosity for transferrin receptor-2 Y250X mutation induces early iron overload. Haematologica 2004;89:359-360.

[16] Hattori A, Wakusawa S, Hayashi H, Harashima A, Sanae F, Kawanaka M, et al. AVAQ 594-597 deletion of the TfR2 gene in a Japanese family with hemochromatosis. Hepatol Res 2003;26:154-156.

[17] Biasiotto G, Belloli S, Ruggeri G, Zanella I, Gerardi G, Corrado M, et al. Identification of new mutations of the HFE, hepcidin, and transferrin receptor 2 genes by denaturing HPLC analysis of individuals with biochemical indications of iron overload. Clin Chem 2003;49:1981-1988.

[18] Girelli D, Bozzini C, Roetto A, Alberti F, Daraio F, Colombari R, et al. Clinical and pathologic findings in hemochromatosis type 3 due to a novel mutation in transferrin receptor 2 gene. Gastroenterology 2002;122:1295-1302.

[19] Mattman A, Huntsman D, Lockitch G, Langlois S, Buskard N, Ralston D, et al. Transferrin receptor 2 (TfR2) and HFE mutational analysis in non-C282Y iron overload: identification of a novel TfR2 mutation. Blood 2002;100:1075-1077.

[20] Hofmann WK, Tong XJ, Ajioka RS, Kushner JP, Koeffler HP. Mutation analysis of transferrin-receptor 2 in patients with atypical hemochromatosis. Blood 2002;100:1099-1100.

[21] Roetto A, Totaro A, Piperno A, Piga A, Longo F, Garozzo G, et al. New mutations inactivating transferrin receptor 2 in hemochromatosis type 3. Blood 2001;97:2555-2560.

[22] Camaschella C, Roetto A, Cali A, De Gobbi M, Garozzo G, Carella M, et al. The gene TFR2 is mutated in a new type of haemochromatosis mapping to 7q22. Nat Genet 2000;25:14-15.

[23] Del-Castillo-Rueda A, Cuadrado-Grande N, Alvarez-Fernandez E, Enriquez-de-Salamanca R, Alvarez-Sala LA, Moran-Jimenez MJ. Mutations in HFE and TFR2 genes in a Spanish patient with hemochromatosis. Rev Esp Enferm Dig 2011;103:379-382.

[24] Majore S, Ricerca BM, Radio FC, Binni F, Consentino I, Gallusi G, et al. Type 3 hereditary hemochromatosis in a patient from sub-Saharan Africa: is there a link between Adrican iron overload and TFR2 dysfunction?. Blood Cells Mol Dis 2103;50:31-32.

[25] Radio FC, Majore S, Binni F, Valiante M, Ricerca BM, De Bernardo C, et al. TFR2-related hereditary hemochromatosis as a frequent cause of primary iron overload in patients from Central-Southern Italy. Blood Cells Mol Dis 2014;52:83-87.

[26] Bardou-Jacquet E, Cunat S, Beaumont-Epinette MP, Kannengiesser C, Causse X, Sauvion S, et al. Variable age of onset and clinical severity in transferrin receptor 2 haemochromatosis : novel observations. Br J Haematol 2013;162:278-281.

**Supplementary Table 2.** **Cloning and sequencing oligonucleotides used in the study**

| Oligonucleotide Name | Purpose | Sequence 5’🡪 3’ |
| --- | --- | --- |
| hs-TFR2_f_14 | Amplification of TfR2 exon 18 from pedigrees 1 and 2* | AGCCCCCAACCCTGACCTGA |
| hs-TFR2_r_14 | Amplification of TfR2 exon 18 from pedigrees 1 and 2* | AGCCACCTCCCTGACCCTGA |
| hs-TFR2_10.2F | Amplification of TfR2 exon 14 from pedigree 2** | GTTAGGCATTGGGGAGAGGT |
| hs-TFR2_10.2R | Amplification of TfR2 exon 14 from pedigree 2** | CAAAGGCCGTGAAGGAATAG |
| Minigene F1 | Genomic DNA PCR | gggatgacaagtttcatgccaagac |
| Minigene R1 | Genomic DNA PCR | ctggggcagggggaggacgtctcac |
| c.1606-8A>G-f | Site-directed TfR2 minigene mutagenesis | TGATCAGTGCCCTTCCCCAGCCCCCAGGTGGATTCTCC |
| c.1606-8A>G-r | Site-directed TfR2 minigene mutagenesis | GGAGAATCCACCTGGGGGCTGGGGAAGGGCACTGATCA |
| G792R-f | Site-directed TfR2 mutagenesis | GCCAATGCGCTTAGCAGGGATGTCTGGAACATT |
| G792R-r | Site-directed TfR2 mutagenesis | AATGTTCCAGACATCCCTGCTAAGCGCATTGGC |
| TfR2-II.2-F | Gene-specific TfR2 primers for RT-PCR | GACAAGTTTCATGCCAAGACC |
| TfR2-II.2-R | Gene-specific TfR2 primers for RT-PCR | GATTGGTGAACACCACCTGTT |
| PT1 sense | RT-PCR for minigene | gtcgacgacacttgctcaac |
| Exon 14 antisense | RT-PCR for minigene | gattggtgaacaccacctgtt |

* PCR conditions for amplification were: denaturation at 94°C, annealing ranging from 66 to 60°C, and extension at 72°C, each step for 30 seconds and for 30 cycles.

** PCR conditions were denaturation at 94°C, annealing ranging from 60 to 56°C, and extension at 72°C, each step for 30 seconds and for 30 cycles.
